# Mental Health in Elite Coaches

**DOI:** 10.1177/19417381231223472

**Published:** 2024-01-21

**Authors:** Laura Baumann, Andres Ricardo Schneeberger, Alan Currie, Samuel Iff, Erich Seifritz, Malte Christian Claussen

**Affiliations:** † Department of Psychiatry, Psychotherapy and Psychosomatics, Psychiatric University Hospital Zurich, University of Zurich, Zurich, Switzerland; ‡ Department of Psychiatry, University of California San Diego, San Diego, California; § Regional Affective Disorders Service, Cumbria Northumberland Tyne and Wear NHS Foundation Trust, Newcastle, UK; ‖ Exercise Sport and Rehabilitation Therapies, University of Sunderland, Sunderland, UK; ¶ Institute for Social and Preventive Medicine, University of Bern, Bern, Switzerland; # Clinic for Depression and Anxiety, Psychiatric Centre Münsingen, Switzerland

**Keywords:** High performance, competitive sports, mental health, coach, sports psychiatry

## Abstract

**Context:**

Coaches play an important role in promoting mental health in elite sports. However, they themselves are exposed to risks affecting their mental health, and their fears and worries are often overlooked. Moreover, it remains unclear how coaches’ mental health affects their athletes’ mental health.

**Objective:**

To create a compilation of the literature on (1) elite coaches’ mental health and (2) how coaches’ mental health influences elite athletes’ mental health. Building on this, recommendations for improving coaches’ psychological well-being should be elaborated upon and discussed.

**Data Sources:**

A literature search was conducted up to November 30, 2021, using the following databases: PubMed, PsycINFO, Scopus, Web of Science, and SportDiscus.

**Study Selection:**

Studies reporting elite coaches’ mental health symptoms and disorders and the influence of elite coaches’ mental health on elite athletes’ mental health were included.

**Study Design:**

Scoping review.

**Level of Evidence:**

Level 4.

**Data Extraction:**

Data regarding elite coaches’ mental health, as well as their influence on athletes’ mental health and performance, were included in a descriptive analysis. The PRISMA guidelines were used to guide this review.

**Results:**

Little research has been done on elite coaches’ mental health disorders, although studies confirm that they do experience, for example, symptoms of burnout, anxiety, and depression. The influence of coaches’ mental health on their athletes is underinvestigated, with research focused mainly on the influence of coaches’ stress.

**Conclusion:**

Knowledge about coaches’ mental health is still limited. Coaches’ poor mental health diminishes coaching performance and might impair athletes’ mental health. Coaches should receive more support, including sports psychiatric care and education on the importance of mental health. This could improve the mental health of both coaches and athletes, and positively affect athlete performance.

In recent years, mental health symptoms and disorders among those in elite and competitive sports have attracted a great deal of attention among both professionals and the general population. However, this attention has focused mainly on mental health problems and mental disorders in athletes.^8,9,10,21,19,27,41,42^ Depression and anxiety disorders, substance use disorders, sleep disturbances, eating disorders, attention deficit hyperactivity disorder (ADHD), and neuropsychiatric deficits after head injuries were found to be highly prevalent among elite athletes.^8,19,41^

While researchers have devoted attention to elite athletes, there has been less focus on other essential professions in the sport setting, such as referees and coaches.^5^ Coaches are also exposed to mental health risks, stress, and escalating pressures and are vulnerable to mental health symptoms and disorders.^1,5,7^ They are likely affected by mental health symptoms and disorders at least as much as the general population.^16^ Sports coaching is challenging, as it involves taking on multiple roles and expectations and includes teaching athletes in several domains, such as physical health and technical, tactical, and social skills.^2,13,14,26,31,41,49^

Coaches’ central focus is to produce competent and successful athletes and to elicit strong performances.^22^ In addition to promoting athletic performance, part of coaching involves fostering the psychological well-being of athletes and ensuring that they are mentally healthy.^17,20,51^ Coaches act as navigators and have the significant responsibility of noticing changes in their athletes’ mental state and behavior. As navigators, they should be able to direct athletes to obtain appropriate support.^4,8,40^ Coaches ought to create a destigmatizing environment and promote positive attitudes toward seeking mental health treatment.^8,41^

Given the importance of the coach’s role within the athletic setting of elite and competitive sports, it is surprising that there is so little research on mental health in this population. Until now, research has focused mainly on stress-related symptoms and burnout among sport coaches,^23^ although initial studies investigating elite depressive symptoms and disorders of coaches in elite sports and in the literature indicate that the psychological strain experienced by coaches needs to be explored.^23-25,35,40^ A better understanding of elite coaches’ psychological well-being is necessary both to provide support and a model of care for this population,^30,40^ and to promote the mental health of athletes.

## Rationale and Objective of the Scoping Review

To our knowledge, no review to date has summarized the mental health symptoms and disorders of coaches in competitive and elite sports. Furthermore, it is largely unknown how athletes’ mental health is affected by their coaches’ mental health. Thus, the present scoping review had 2 main purposes:

1. To compile knowledge on the mental health symptoms and disorders of coaches in elite and competitive sports; and
2. To identify the influence of the mental health of coaches on the mental health of athletes in elite and competitive sports.

Considering the important role of coaches for athletes and their vulnerability to mental health problems, strategies to support coaches’ mental health are necessary. Therefore, based on the available literature and our findings, recommendations for improving coaches’ mental health should be elaborated upon and discussed, including the ways in which they relate to athlete performance.

## Methods

The databases PubMed, PsycINFO, Scopus, Web of Science, and SportDiscus were used to identify relevant studies with the following keyword string: (coaches OR trainer) AND (elite sports OR competitive sports) AND (mental health OR mental health symptoms OR mental health disorders). Included in the search were all studies in English or German without a restriction on publication date. All study designs were included. The International Olympic Committee (IOC) defines elite athletes as those competing at professional, Olympic, or collegiate levels (eg, National Collegiate Athletic Association Division I). We used this definition to enable comparison of the studies.^41^ As a synonym for elite athletes, we accepted the terms “high-performance” or “high-level” athletes. Therefore, the coaches considered for this study needed to train athletes competing at those levels. Articles that focused on nonelite athletes (eg, club level, NCAA Division II and III) or an unclear level of competition were not included. There was no restriction regarding the age of athletes and coaches. Years of experience and coaching success were not relevant for inclusion. Articles that focused mainly on elite athletes’ mental health, without any information concerning coaches’ mental health or their influence on elite athletes, were excluded. Studies with a focus on sport psychology - for example, how to develop mental strength or toughness - were not included.

**Figure 1.**
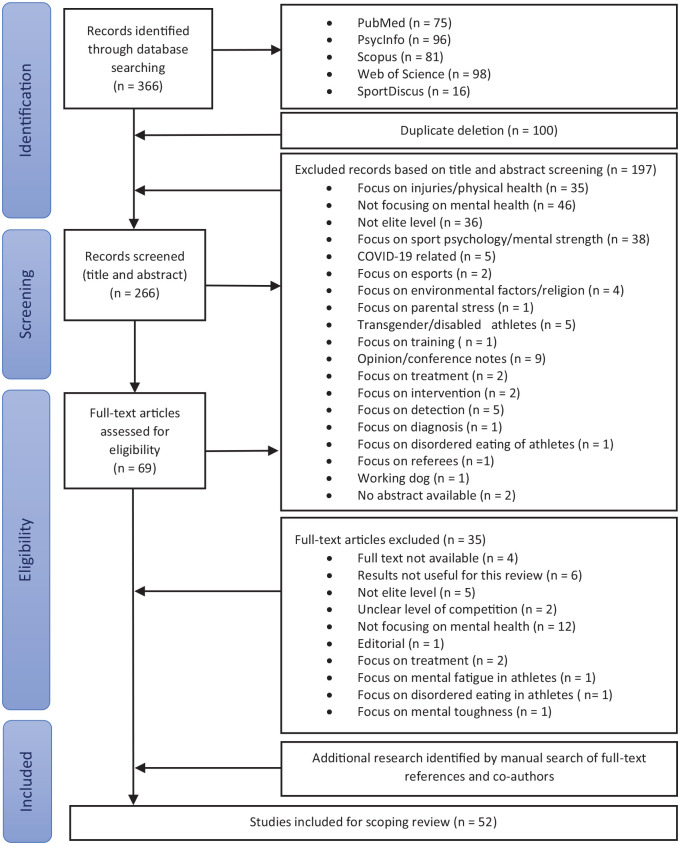
Flow diagram summarizing the study selection criteria.

First, 1 reviewer screened the titles and abstracts and used the above criteria to exclude articles that were not relevant. Studies that did not provide sufficient information in the title or abstract were included for full text review. After excluding studies based on the titles and abstracts, the reviewer examined the remaining articles based on the full texts. Articles that remained unclear were discussed with another reviewer. The references of the obtained full-text articles were screened to prevent missing relevant studies. Figure 1 presents an overview of the search procedure.

### PRISMA Guidelines and Scoping Review

PRISMA (Preferred Reporting Items for Systematic Reviews and Meta-Analyses) guidelines were used to guide this review; all mandatory items that relate to a scoping review were applied.^50^ We decided to write a scoping review instead of a systematic review because our first aim was to provide an overview regarding coaches’ mental health and we did not perform an assessment of risk of bias or methodological limitations.^33^

## Results

In total, 366 publications were identified using the named databases; 100 of these citations were duplicates (Figure 1). Of the remaining articles, 197 were excluded because they had inappropriate titles and abstracts. Therefore, 69 full texts were screened for eligibility, of which 35 were excluded for the following reasons: full text not available (n = 4), results not useful for this review (n = 6), not elite level (n = 5), unclear level of competition (n = 2), not focused on mental health (n = 12), editorial (n = 1), focus on treatment (n = 2), focus on mental fatigue among athletes (n = 1), focus on disordered eating in athletes (n = 1), and focus on mental toughness (n = 1). Another 18 articles were included after searching the references and conducting additional research. Altogether, 52 articles were identified as relevant for this scoping review. The study design varied, although most were qualitative studies. Most studies were based on questionnaires that could measure mental health symptoms, but not mental disorders, as clinical diagnoses can be made only by a clinician, such as a psychiatrist.

### Mental Health Symptoms and Disorders of Coaches

The collected data were summarized and classified according to the International Classification of Diseases 10th/11th Revision (ICD-10/11) codes.^15,52,53^ Listed are only those mental health symptoms and disorders mentioned in the publications. A large number of mental disorders among elite coaches were not accounted for as there was no research on them, such as ADHD, obsessive-compulsive disorder (OCD), bipolar disorders, psychotic disorders, personality disorders, sleep disturbances, and eating disorders.

#### Substance Use Disorder:

Very few studies have investigated substance abuse among elite coaches. One study of 12 elite coaches in the United Kingdom (UK) revealed that drinking too much alcohol was a way for coaches to distract themselves when experiencing stress.^37^ Another paper reported a single interview with an elite coach and his wife from the UK, in which the coach used alcohol to handle his depressive symptoms and fear of failing since expectations were high and the pressure to deliver was immense.^43^

#### Depression:

A recent cross-sectional survey found the prevalence of moderate depressive symptoms among elite coaches in New Zealand to be 14%. This prevalence rate is comparable with that of the general population in New Zealand but lower than that of New Zealand’s elite athletes (20%).^25^ The depressive symptoms were associated with major life stressors such as facing retirement and a history of family mood disorders.^25^ Prolonged job insecurity and ongoing stress were found to be factors associated with depressive symptoms.^3,37^

#### Anxiety:

One case study discussed an elite coach who experienced anxiety symptoms concerning the failure of their athletes to qualify for a competition or not reaching their usual performance level.^24^ Moreover, job insecurity is correlated with high levels of anxiety.^3^

#### Burnout:

Burnout is one of the mental health problems most examined among elite coaches.^45^ Studies revealed that contributory factors include concerns about athletes, coaches’ responsibility to athletes, the consequences of sport status, competition and training preparations, sacrificing personal time, and isolation.^14,45^ Female coaches and coaches who lack coping strategies to deal with the performance culture or tools to enhance recovery are more likely to suffer from burnout.^1,45^ In addition, an obsessive passion for coaching and setting a high value on the pursuit of winning and rewards are reported to be linked to higher risks for emotional exhaustion.^1,45^

### Influence of Elite Coaches’ Mental Health on Athletes’ Mental Health

Research did not specifically investigate the influence of elite coaches’ mental health on elite athletes’ mental health, but it revealed how coaches’ behavior impacts their athletes. When a coach exhibits controlling behavior, their athletes are more likely to develop anxiety symptoms, which are in turn a risk factor for burnout.^11^ A study of female soccer players in Germany demonstrated how coaches’ behavior impacts the emotional status of players. Conflicts with coaches, followed by athletes’ injuries, were the reasons cited most commonly for low moods among athletes.^39^ A lack of support from coaches was also associated with athletes’ low moods and negative feelings,^39,46^ while athletes can be negatively affected by coaches’ stress.^14,25,37,48^

### How Elite Coaches’ Mental Health Affects Their Performance

No study has directly examined how coaches’ mental health symptoms and disorders influence their ability to do their job, including all of its necessary tasks. Three studies have, however, reported on the effects of stress on coaching performance. Coaches’ stress results in changes to their behavior, appearance, and style of communication.^47^ Athletes are able to recognize when their coaches experience stress,^29^ and perceive their coaches to be less effective when stressed.^47^ Coaches’ stress leads to reduced time for feedback and anger toward athletes, which contributes to athletes losing confidence, becoming angry at themselves, and performing less well.^37^

## Discussion

Little research has been done on the mental health and mental health disorders of elite coaches, although studies confirm that they do experience mental health symptoms and disorders, especially symptoms of burnout,^1,14,45^ anxiety,^1,3,24^ and depression.^25,43^ However, several mental health symptoms and disorders - personality disorders, for example - have not been investigated in elite coaches. This is remarkable, as it is known that coaches who are experiencing stress probably negatively influence both the well-being and performance of athletes.^1,35,37^

### Coaches’ Mental Health and Its Influence on Athletes’ Mental Health and Performance

Coaches’ mental health and its influence on athletes is underinvestigated. Research has examined primarily the consequences of ongoing stress and its influence on athletes. The causes of coaches’ stress include recruitment, pressure to win, high demands, organizational management, and job insecurity.^3,14,23,24,35,45,47^ Stress changes coaches’ behavior and their style of communication,^47^ which leads to reduced time for feedback and eventually results in athletes’ worsened performance.^37^ Coaches themselves are aware that their negative responses to stress are projected onto athletes.^37^

The behavior of a coach may be influenced strongly by their mental condition. Their psychological well-being positively predicts behavior that is supportive of athlete autonomy as opposed to controlling behavior toward athletes.^35^ In contrast, symptoms of poor mental health or well-being might lead coaches to distance themselves from their athletes, making it more difficult to maintain a healthy coach-athlete relationship.^34^ Maintaining a good coach-athlete relationship is especially important in talent development, where it is key for athletes’ well-being,^40,44^ and because adolescent athletes are more sensitive to coaches’ opinions.^8^ Besides promoting athletic performance, part of the coach’s role is to foster athletes’ personal, emotional, and psychosocial development,^2,22,32^ the importance of which should not be underestimated. For these reasons, we believe that coaches who suffer from mental health symptoms and disorders lack the ability to consistently achieve their best coaching performance and to effectively fulfil all coaching tasks.

Coaches’ mental health should be supported, and clubs and sport organizations should show an interest in the appropriate management of coaches’ health and well-being. We believe that the results of such interest would be improved coaching performance and, eventually, the improved mental health and performance of athletes. However, as coaches have a right to be healthy themselves, the focus should be on mental health, and not on improving performance. The main objective is to provide support for mental health symptoms and, if necessary, the treatment of mental health disorders, which, if they remain unrecognized or are inadequately treated, can lead to more serious health problems. An increase in performance is a desirable byproduct of good treatment and care for mental health problems.

### Approaches to Support Coaches’ Mental Health

Although previous research articulated an increasing awareness that coaches in sport settings can be vulnerable to mental health symptoms and disorders, to date there are no frameworks to support coaches’ mental health.^5,18^ Despite the need for mental health interventions, strategies to better support coaches are lacking and still need to be developed.^5,30^ Possible solutions involve the provision of mental health education, monitoring of well-being, and the removal of barriers to offering appropriate assistance, including mental health services (Table 1).^25^

**Table 1.** Approaches to promote coaches’ mental health

Target Audience	Suggested Actions
Coaches	• Education programs^38^ • Exchange with other coaches^14,37,38^ • Coaches as mentors^38^ • Mental health in coaching qualification and training
Mental health professionals and sport physicians	• Basic care through sport physicians^12^ • Annual PPE • Regular appointments with coaches • Screening for the most relevant mental health disorders • Diagnosis, therapy and aftercare^12^ • More education, including supervision on sport-specific mental health concerns
Clubs and sport federations	• Educate and provide training about mental health and disorders • Acknowledge the issue of mental disorders and the vulnerability of coaches in elite sports • Raise awareness of mental health and disorders • Create a destigmatizing environment • Offer abused athletes a place to go • Design tools to support the mental health of coaches • Implement and monitor policies for the protection of athletes

PPE, preparticipation evaluation.

Coaches’ mental health should be discussed and could be improved by educational programs, seminars, and mental skills training.^25,37^ Current educational programs consist mainly of strategies to promote athletic performance but should in future also focus on coaches, containing basic information on mental health, stress control, and other key psychological skills.^38^ In addition, experienced coaches can guide younger colleagues by sharing their knowledge and thus enabling less experienced coaches to manage stress and poor mental well-being.^38,14,37^ Experienced coaches will also benefit from peer support and exchanges with other coaches.^38^ We suggest that mental health should form part of the syllabus in coaching qualification training, with the aim of increasing coaches’ mental health literacy and imparting information concerning the possibilities of getting help.

The monitoring of psychological well-being is important and should be supervised by appropriately qualified clinicians.^30^ Mental healthcare services include psychiatrists, psychologists, and psychotherapists, as well as clinical social workers and primary care physicians, including sports physicians with core competencies.^9^ Research has shown that working alongside sport psychologists helps coaches to better cope with stress,^37^ and has even helped them to develop into Olympic coaches.^38^ Assessment, diagnosis, therapy, treatment, and aftercare for psychiatric disorders require experienced mental health clinicians, such as psychiatrists, sports psychiatrists, and clinically trained psychologists and psychotherapists.^12,30^ Greater availability of these professionals in sports associations, clubs, and teams could reduce barriers to accessing mental health services, as services will be used if freely available.^30^ Moreover, regular appointments and an established relationship facilitate the early recognition, intervention, and management of mental health symptoms and disorders.^8,43^ Responsibility for basic mental healthcare can also be assumed by primary care physicians and sport physicians who have received appropriate continuing education, optimally in collaboration with a psychiatrist or sports psychiatrist.^12^ In addition, we advise that a preparticipation evaluation should be offered to both coaches (obligatory from a certain level) and athletes, which should include a screening for mental health symptoms and disorders. In addition, coaches should also have low-threshold access to sports psychiatry, examinations, and treatment. Unfortunately, currently only a small number of psychiatrists specialize in the field of sport, which represents a gap in mental healthcare provision for athletes and coaches. To fulfil the prerequisites for specializing in this area, academic institutions should broaden their educational programs to enable practitioners and psychiatrists to specialize in subspecialties that are specific to sports.

Sport staff should be educated about mental disorders. With the guidance of mental health professionals, key support staff can develop competencies to identify mental symptoms and the early signs of mental disorders in coaches.^43^ Sport organizations and sport clubs must be aware of their duty of care and should implement and promote places and structures where sport staff can ask for advice.^6^ These support structures should also be accessible to athletes, especially as athletes work closely with coaches and may notice inappropriate behavior and poor mental well-being early on. In addition, it is the responsibility of the federations and clubs to create a working environment in which coaches do not feel stigmatized when asking for help.^43^ Sports associations and clubs should also acknowledge specific problems, such as job insecurity, and initiate communication to reduce tensions and build trust.^36^

National and international sport federations and clubs need to further acknowledge coaches’ mental health and the stressors that are specific to their job, as well as working to remove the stigma associated with mental health. Coaches who make their problems public may be discredited, as a coach suffering from mental disorders does not fit the stereotype of a self-confident and strong personality.^43^ In addition, governing bodies and sport clubs should offer psychiatric help, which can be discussed during competitions. A case study showed that more support from governing bodies, such as Olympic committees and national sports federations, would help coaches dealing with mental health symptoms.^24^ Besides raising awareness, sport federations and sport clubs have the responsibility of developing strategies and guidelines.^6^ Exemplary, therefore, is the tool “SMRTH-1,” which was designed by the International Olympic Committee for the early detection and support of mental health symptoms among elite athletes.^20^ We suggest the development of similar tools specifically for coaches to ensure early detection and access to high-quality interventions. Furthermore, sport organizations need to develop mental health literacy strategies that can be integrated into organizational structures.^18^ Such literacy is too often seen as the responsibility of the individual person, while it should be the role of organizations to facilitate this.

## Limitations

This scoping review has several limitations, one of which is the great heterogeneity among the included studies. Only a small number of studies focused exclusively on the mental health of coaches. Many studies were based on questionnaires, which can measure mental health symptoms but do not necessarily detect mental disorders. Due to this type of data acquisition, mental health symptoms were mainly self-reported and could not be classified according to either the ICD-10/11 or The 5th Edition of Diagnostic and Statistical Manual of Mental Disorders (DSM-5) codes. Furthermore, certain mental disorders (eg, ADHD, OCD) among elite coaches were left out as they were not represented in research. Moreover, most studies were conducted with a small sample of coaches. Also, the studies were conducted in different countries, in different sports and in many cases without reporting the gender of the coaches or athletes. As a consequence, it is difficult to draw conclusions and make comparisons across several sports (eg, team vs individual sports) and between genders and age groups. In addition, the definition of an elite athlete was heterogeneous since we accepted the synonyms high-performance and high-level athlete. Lastly, the risk of bias and the quality of evidence was not checked, as would have been the case for a systematic review.

## Implications for future research

The scoping review indicates that elite coaches are at risk of suffering from mental health symptoms and disorders for several reasons.^1,24,25,43,45^ More research on coaches’ mental health is needed to obtain a better understanding of the extent to which this specific population is affected. Future studies should include clinical interviews, conducted by, for example, psychiatrists, so that a diagnosis according to the ICD-10/11 or Diagnostic and Statistical Manual of Mental Disorders, Fifth Edition (DSM-5) codes can be ascribed and the prevalence of disorders (rather than simply symptoms) established. This survey method is time consuming, expensive, and requires certain qualifications and competencies of the interviewer, which may be the main reason why there are so few such studies. Many mental disorders - such as ADHD, OCD, personality disorders, bipolar disorders, and psychotic disorders - have not been researched and should also be included in future studies. Longitudinal studies that follow the careers of coaches will impart more information about the development of mental health problems and disorders. Studies conducted with a large cohort that can be followed over a long period of time would permit exploration of the influence of competitions such as the Olympics and world championships. Moreover, they would allow for the examination of a coach’s mental health at different timepoints, such as the preseason and before and after competitions. Future studies should also examine sports below the elite level, as it is likely that nonelite coaches also experience mental health issues, which could in turn affect their athletes.

Further investigations of the potentially negative influence of coaches’ mental health on their athletes, as well as the coach-athlete relationship, are also needed.^28^ Moreover, research should address the impact of coaches’ mental health problems and disorders on their coaching performance. Since vulnerability and psychiatric disorders are often perceived as weaknesses in elite sports, education about mental health and the development of strategies that foster coaches’ well-being and the involvement of mental health professionals are necessary.

## Conclusion

Knowledge about mental health symptoms and disorders among elite-level sports coaches is still limited. Nevertheless, the available literature has revealed that coaches do suffer from mental health concerns such as burnout, anxiety, and depressive symptoms. Because of the close working alliance between a coach and an athlete, coaches’ mental health might impair elite athletes’ mental health and worsen their performance. This scoping review illustrates the urgent and important need for longitudinal studies that examine coaches’ mental health and psychological well-being and how these influence elite athletes’ mental health and performance. Sports organizations and sports federations should establish a minimum standard of care and implement specific management strategies to address the mental health needs of this population. Furthermore, sports psychiatric care and education on the importance of mental health are necessary to support both coaches and athletes.
